# Does telecare prolong community living in dementia? A study protocol for a pragmatic, randomised controlled trial

**DOI:** 10.1186/1745-6215-14-349

**Published:** 2013-10-23

**Authors:** Iracema Leroi, John Woolham, Rebecca Gathercole, Robert Howard, Barbara Dunk, Chris Fox, John O’Brien, Andrew Bateman, Fiona Poland, Peter Bentham, Alistair Burns, Anna Davies, Kirsty Forsyth, Richard Gray, Martin Knapp, Stanton Newman, Rupert McShane, Craig Ritchie

**Affiliations:** 1 Institute of Brain, Behaviour and Mental Health, University of Manchester, Jean McFarlane Building, 3rd floor, Oxford Rd, Manchester M13 9PL, UK; 2 Faculty of Health & Life Sciences, Charles Ward Building, Coventry University, Coventry CV1 5FB, UK; 3 Department of Old Age Psychiatry, King’s College London, Institute of Psychiatry, De Crespigny Park, Box 070, London SE5 8AF, UK; 4 Department of Old Age Psychiatry, Institute of Psychiatry, De Crespigny Park, Box 070, London SE5 8AF, UK; 5 South London & Maudsley NHS Foundation Trust, 115 Denmark Hill, The Maudsley, London SE5 8AZ, UK; 6 School of Medicine, Health Policy and Practice, University of East Anglia, Norwich, Norfolk NR4 7TJ, UK; 7 Department of Psychiatry, University of Cambridge, Level E4, Box 189, Addenbrookes Hospital, Hills Road, Cambridge CB2 0QQ, UK; 8 Oliver Zangwill Centre for Neuropsychological Rehabilitation, Princess of Wales Hospital, Ely CB6 1DN, UK; 9 School of Allied Health Professionals, University of East Anglia, Norwich, Norfolk NR7 4TJ, UK; 10 The Barberry Centre, 25 Vincent Drive, Birmingham B15 2FG, UK; 11 School of Community Based Medicine, University of Manchester, Jean McFarlane Building, 3rd floor, Oxford Rd, Manchester M139PL, UK; 12 School of Community and Health Science, City University, University Building A224, St John St, London EC1V 0HB, UK; 13 Queen Margaret University, Edinburgh EH21 6UU, UK; 14 Clinical Trial Service Unit, Richard Doll Building, Old Road Campus, Roosevelt Drive, Oxford OX3 7TF, UK; 15 Personal Social Services Research Unit, London School of Economics and Political Science, Houghton St, London WC2A 2AE, UK; 16 School of Community and Health Sciences, City University, Northampton Square, London EC1V 0HB, UK; 17 Oxfordshire & Buckinghamshire Mental Health NHS Foundation Trust, Warneford Hospital, Old Road, Headington, Oxford OX3 7JX, UK; 18 Centre for Mental Health, Imperial College London, Claybrook Centre, Charing Cross Campus 37 Claybrook Rd, London W6 8LN, UK

**Keywords:** Assistive technology, Telecare, Dementia, Randomised control trial, Independence, Community living

## Abstract

**Background:**

Assistive technology and telecare (ATT) are relatively new ways of delivering care and support to people with social care needs. It is claimed that ATT reduces the need for community care, prevents unnecessary hospital admission, and delays or prevents admission into residential or nursing care. The current economic situation in England has renewed interest in ATT instead of community care packages. However, at present, the evidence base to support claims about the impact and effectiveness of ATT is limited, despite its potential to mitigate the high financial cost of caring for people with dementia and the social and psychological cost to unpaid carers.

**Method/design:**

ATTILA (Assistive Technology and Telecare to maintain Independent Living At Home for People with Dementia) is a pragmatic, multi-centre, randomised controlled trial over 104 weeks that compares outcomes for people with dementia who receive ATT and those who receive equivalent community services but not ATT. The study hypothesis is that fewer people in the ATT group will go into institutional care over the 4-year period for which the study is funded. The study aims to recruit 500 participants, living in community settings, with dementia or significant cognitive impairment, who have recently been referred to social services.

Primary outcome measures are time in days from randomisation to institutionalisation and cost effectiveness. Secondary outcomes are caregiver burden, health-related quality of life in carers, number and severity of serious adverse events, and data on acceptability, applicability and reliability of ATT intervention packages. Assessments will be undertaken in weeks 0 (baseline), 12, 24, 52 and 104 or until institutionalisation or withdrawal of the participant from the trial.

**Discussion:**

In a time of financial austerity, CASSRs in England are increasingly turning to ATT in the belief that it will deliver good outcomes for less money. There is an absence of robust evidence for the cost-effectiveness and benefit of using assistive technology and telecare. The ATTILA trial meets a pressing need for robust, generalisable evidence to either justify continuing investment or reappraise the appropriate scale of ATT use.

**Trial registration:**

Current Controlled Trials ISRCTN86537017

## Background

There are approximately 800,000 people with dementia in the UK [1], many of whom will require accommodation in nursing or residential care homes when their illness has progressed to the point at which they can no longer live safely and independently in their own homes. It has been estimated that over the next 2 decades the number of people aged 85 and over will increase by two-thirds [2]. Over half of all users of adult social care services are aged 65 and over [3], and a steep rise in the numbers of people living with dementia is expected over the next few decades. The financial cost of caring for people with dementia is considerable [1], as is the social and psychological cost to unpaid carers (generally a relative or friend, subsequently referred to as ‘carers’). Carer breakdown is a common reason for the unplanned admission of older people (many of whom will have dementia) into permanent nursing or residential care [4].

Living Well with Dementia, the theme of the 2009 National Dementia Strategy for England [5], involves helping people with dementia retain their independence while living in their own homes as well as to maintain their quality of their life. People living with dementia who move from their own homes into institutional care often experience a loss of independence and quality of life. In order to minimize this possibility, the National Health Service (NHS) and Councils with Adult Social Services Responsibilities (subsequently referred to as CASSRs) in England aim to support people with dementia to live safely in their own homes for as long as possible.

Assistive technology and telecare (ATT) offer relatively new means of delivering care and support to people with social care needs by helping to manage risks facing older people with dementia who wish to remain living independently at home. Sensors to detect falls, floods, or the presence of gas from an unlit appliance in someone’s home, for example; passive monitoring, for example, sensors placed within a home environment to detect movement; and alerting devices to relay information from the person’s home to a remote site such as a call centre are claimed to support the independence of people with social care needs, to reduce the burden on unpaid carers [6-10] and to save CASSRs money. By addressing risks associated with independent living for people with dementia, ATT has claimed to help reduce the need for community care, prevent unnecessary hospital admissions, and delay or prevent admission into residential or nursing care. [7,11-13] The evidence to support such claims is limited and based largely on qualitative evidence or uncontrolled quantitative studies [14,15]. There is, therefore, an urgent need to provide evidence to inform decisions about whether to provide ATT in the homes of people with dementia. ATTILA is designed to answer questions about the efficacy and cost-effectiveness of ATT, with relevance for those who commission and provide care for people with dementia.

### Research evidence

The first use of electronic ATT in the UK, in the 1990s, was to provide support for people with dementia and their carers [11,16-19]. Within a decade, interest in ATT has developed from a fringe interest for a handful of enthusiasts to a multi-million pound industry commanding government support, a Department of Health strategy [20] and, increasingly, the use of ATT in CASSR settings as a mainstream service (see, for example [19]). However, as interest in ATT has increased, the specific focus on its application for those living with dementia has diminished [8]. The performance indicators that followed the Preventive Technology Grant (given to CASSRs in 2008 by the Department of Health [20]) were intended to promote the widest possible use of telecare. The Department of Health did not, however, offer a clear indication of what this grant was supposed to ‘prevent’. Although Woolham [7] has drawn attention to the cost-effectiveness of telecare for people with dementia by closely matching ATT with assessed need, and thereby preventing the need for more expensive forms of care, Poole [21] has argued that CASSRs should see ATT as a long-term investment, deploying it at an early stage without expecting immediate savings. This has contributed to a situation in which CASSRs have implemented ATT across several different care groups without always referring to the needs of the specific groups, such as people with dementia. The current economic situation, as well as a significant reduction in government CASSR funding, has led to increasing numbers of CASSRs developing an interest in ATT. Some have developed local strategies to use it, whilst others already have well-developed ATT services that can be deployed alongside, or instead of, non-institutional forms of support, often known as ‘community care’ within the UK.

Despite growing ATT use, the evidence to support its use is limited. The Whole System Demonstrator study (WSD) was funded by the Department of Health in 2008 to investigate the impact and effectiveness of ATT in England [22-27], Steventon A, Bardsley M, Billings J, Dixon J, Doll H, Beynon M, Hirani S, Cartwright M, Rixon L, Knapp M, Henderson C, Rogers A, Hendy J, Fitzpatrick R, Newman S: The effect of telecare on the quality of life and psychological well-being of elderly recipients of social care over a 12 month period – the whole systems demonstrator (WSD) cluster randomised trial, forthcoming, Henderson C, Knapp M, Fernández JL, Beecham J, Hirani S, Beynon M, Cartwright M, Rixon L, Doll H, Bower P, Steventon A, Rogers A, Fitzpatrick R, Barlow J, Bardsley M, Newman S, Whole System Demonstrator Evaluation Team: Cost-effectiveness of telecare for people with social care needs: the whole systems demonstrator cluster randomised trial, forthcoming]. However, individuals with dementia were not specifically included. This, together with the relative dearth of dementia-specific studies in ATT, means that a significant gap remains in our evidence. Although there are relatively large numbers of qualitative studies, audits and service evaluations, there are few studies with sufficient rigour and appropriate design to offer any degree of generalisability [15] or agreement about how ‘success’ can be measured [28]. One study has suggested that, when used appropriately, ATT is highly cost-effective. Unfortunately, limitations in design and methodology constrain the generalisability of this study’s findings [19]. Evidence from a well-designed study such as ATTILA may therefore also be used to guide future policy direction.

### Policy guidance

The Department of Health in England’s ‘Building Telecare’ strategy in 2005 [20] provided generic advice to CASSR. As part of this strategy, a Preventive Technology Grant, which CASSRs were required to spend on developing local ATT services, was made available. England’s 2009 National Dementia Strategy [14] recommended a ‘watching brief’ for emerging evidence of the impact of telecare, stating:

*However, with respect to more recent innovations, this is not an area where the strategy is able at this time to make specific recommendations. Instead, central, regional and local teams should keep in touch with initiatives in the areas of housing and telecare and make appropriate commissioning decisions as data become available, for example from the Department’s large-scale field trials of telecare and assistive technology.* Department of Health (2009) p. 56

### Aim of the study

The aims of the ATTILA trial are to test the following hypotheses:

• That the application of ATT will significantly *extend the time* that people with dementia can continue to live independently and safely in their own homes.

• That ATT interventions are *cost-effective* in the management of risk and maintenance of independence in people with dementia living in their own homes.

• That provision of ATT interventions to people with dementia living at home will significantly *reduce the number of incidents* involving serious risks to safety and independent living, particularly those involving acute admissions to hospital,

• That ATT interventions will *reduce burden and stress* in family and other unpaid carers and *increase quality of life* for people with dementia.

### Objectives

These hypotheses will be tested by the following primary and secondary objectives:

#### Primary objectives

To establish whether ATT assessments and interventions *extend the time* that people with dementia can continue to live independently in their own homes and whether this is *cost-effective*.

#### Secondary objectives

(1) To establish whether these technologies can significantly *reduce the number of incidents* involving serious risks to safety and independent living, including acute admissions to hospital.

(2) *To reduce burden and stress* in family and other unpaid carers; and *increase quality of life* for people with dementia and their unpaid carers.

(3) To collect qualitative and quantitative data from people living with dementia, their paid and unpaid carers, and members of the NHS and CASSR teams about their *experience of using* these technologies.

## Methods

### Design

ATTILA is a pragmatic randomised controlled trial over 104 weeks. Blinding will not be undertaken as it is not feasible. It will be conducted in the homes of people living with dementia and who are eligible to receive a package of social care through CASSRs. The trial will compare outcomes in two groups of participants randomised to one of two study arms: either (1) receiving an ATT needs assessment, followed by the installation of ATT devices and response services which will be deployed by the CASSR, or (2) receiving a semi-structured ATT needs assessment, followed by the installation of an ATT package restricted to only smoke and carbon monoxide detectors and a pendant alarm if indicated, also arranged by CASSRs. The key outcomes will be *time to institutionalisation* and *cost-effectiveness* of the intervention.

The trial is not funded to source, assess the need for or deploy ATT. Our approach is therefore to work alongside CASSRs that have been charged by the Department of Health with responsibility for establishing and developing local ATT services.

The study has been approved by the NHS Health Research Authority National Research Ethics Committee (REC reference number 12/LO/186) and is registered with the ISRCTN (http://www.controlled-trials.com/ISRCTN86537017).

### Planned inclusion and exclusion criteria

#### Inclusion criteria

Participants will be people with dementia or evidence of cognitive difficulties significant enough to suggest the presence of dementia without having been formally diagnosed as having dementia, both with and without capacity. This will include those with young or later-onset dementia. Additionally, all participants must fulfil the following criteria:

• Meeting England’s eligibility criteria for access to social care services FACS (Fair Access to Care Services);

• Living in an ordinary community dwelling (including sheltered and very sheltered accommodation);

• Have a working telephone line in their home.

#### Exclusion criteria

People ineligible to take part will be those:

• Receiving an ATT intervention already (excluding the sole provision of smoke, carbon monoxide or pendant alarms) or where ATT has previously been provided but has not been used;

• Being unlikely to comply with follow-up, e.g. because of an unstable medical condition;

• Participating in another clinical trial involving an intervention for dementia;

• Having an urgent need for a care package due to immediate and severe risks to themselves or others.

### Recruitment/consent procedures

There will be several routes for participant recruitment. NHS services (mental health or otherwise) will likely be the first-line sources for recruitment. We will extend recruitment beyond the NHS to local social services and include all people with dementia assessed through the FACS processes (as did the WSD) [23]. Those referred from the NHS will have to meet FACS eligibility and those referred via CASSRs will either have a diagnosis of dementia or evidence of severe memory impairment.

Participating CASSRs will, after assessing FACS eligibility, ask all FACS-eligible referrals who may meet the other ATTILA trial eligibility criteria if their contact details can be made available to a named individual in a local ATTILA research team. Once identified, they will be registered with the ATTILA Study Office. A member of the local research team will contact this person and recruit them according to the trial standard operating procedures, followed by the appropriate randomisation procedures.

Consent will be sought using a defined protocol that meets the requirements of England’s Mental Capacity Act (2005), and where appropriate, data sharing agreements will previously have been agreed with the CASSR concerned to ensure that the transfer of personal data from the CASSR to the research team is lawful. If consent is not given, the individual will not be recruited and all of their personal data will be removed from research team records and destroyed.

For FACS-eligible participants who come from CASSRs, referrers will contact the local ATTILA study centres, which will arrange for a researcher to visit the prospective participant to explain the study and check eligibility to take part in the trial. If there is an urgent need for community or other forms of care or support services, then consent will be sought at that visit so that the prospective participant can be immediately randomised. If an urgent need for social services is not apparent, then information about the study will be left with the prospective participant and the researcher will return 24 h later to obtain consent to participate in the study. The participant will then be randomised. After randomisation, assessment for ATT and for provision of ATT services (within limits set by randomisation) will be left entirely to the local social services authority concerned. Participants who come from the NHS will only be eligible once FACS eligibility has been established by the CASSR.

### Assessments

Assessments will be undertaken by local investigators employed by the ATTILA team in weeks 0 (baseline), 12, 24, 52 and 104. All local investigators are researchers with clinical experience.

Following an assessment of mental capacity (and therefore ability to consent), confirmation of eligibility to take part, the consent process and randomisation, both groups will receive an initial assessment using the following scales: the Bristol Activities of Daily Living Scale (BADLS) [29], Standardised Mini-mental State Exam (SMMSE) [30,31], the Client Services Receipt Inventory (CSRI) [32], The Model of Human Occupation Screening Tool (MOHOST) [33] and the EuroQol EQ-5D [34]. Unpaid carers of participants will be assessed over the same intervals using the Zarit Burden Inventory (ZBI) [35], The Centre for Epidemiological Studies Depression Scale (CES-D10) [36], the State Trait Anxiety Inventory (STAI-6) [37], the Short Form Health Survey (SF-12v2) [38] and the Carer Technology Acceptance Questionnaire (SUTAQ) [39]. All of the outcome measures have established validity and high levels of inter- and intra-rater reliability. No pre-testing before randomisation is possible because of concerns by local authority hosts that this will delay the deployment of care and/or telecare to people who may have urgent needs for support.

### Primary outcome measures

#### Time in days from randomisation to institutionalisation

This is defined as the permanent transition of the individual from living in their own home to nursing or residential care home or to admission to an acute care facility that results in permanent placement in a residential care or nursing home.

#### Cost-effectiveness

Costs will be calculated by attaching nationally applicable unit cost measures to health and social service use (collected at intervals stated above for each participant using a modified version of the CSRI), using national figures taken from the Personal Social Services Research Unit (PSSRU) annual compendium [40] where available. The costs of ATT will be calculated anew for those services provided, and in this we will be guided by methods and experience gained in the WSD trial of telecare and telehealth [25]. *Data on carer time and task inputs* will come from the CSRI, and will be valued using two approaches (replacement wage and opportunity cost) and then comparing the results in sensitivity analyses.

*Cost-effectiveness analyses* will be of two types to take into account the complication that the primary outcome (time to institutionalisation) is itself partly an indicator of resource use. Each type of analysis will then be conducted from each of two perspectives: for the ‘formal’ health and social care system (which is, for example, the perspective employed by the National Institute for Health and Care Excellence in England and Wales to appraise health technologies as a basis for producing clinical guidelines [41]) and for society as a whole (which would then additionally include the value of carer time and any productivity losses from disrupted employment, either for the person with dementia or the carer).

The first type of cost-effectiveness analysis will measure costs only up to the point at which a study participant goes into a care home or hospital and not beyond (‘community costs’) and will then examine cost-effectiveness in achieving the primary outcome (days from randomisation to institutionalisation in the two year period). This analysis will show the incremental cost of community-based support for each additional institutional day avoided. The second type of analysis will measure costs for the whole 2-year period, including costs of care home and hospital stays (‘total costs’), and will then examine cost-effectiveness where the outcome is EQ-5D change, also over the 2-year period. We will also use the EQ-5D to generate Quality-Adjusted Life Year (QALY) measures by attaching societal weights [42].

We will compute incremental cost-effectiveness ratios and plot cost-effectiveness acceptability curves, which will be generated from the net benefit approach and using bootstrap regression for a range of values of willingness to pay for the corresponding outcomes. In each case, we will also be able to carry out these analyses at 24 and 52 weeks.

### Secondary outcome measures

#### Burden in carers

We will measure both burden associated with care-giving and levels of psychological distress among the principal carers of participants at baseline, 12, 24, 52 and 104 weeks using the 22-item short version of the ZBI [35].

#### Quality of life

We will measure health-related quality of life in carers using the SF-12v2 [38].

#### Number and severity of serious adverse events

As in any trial, serious adverse events (requiring GP or hospital care) will be recorded and reported. Details of how we propose to do this are presented below in Sect. 6, which deals with safety monitoring procedures.

#### Quantitative and qualitative data

Data on acceptability, applicability and reliability of ATT intervention packages will be collected using the SUTAQ. This questionnaire is currently being validated using data from the WSD. We will collect unpaid carers’ experiences, which will provide examples of ways in which their lives, well-being and care-giving roles may have been enhanced and/or undermined by the use of these technologies. Variation in their experiences and the reasons for this variation will be explored through semi-structured interviews with individuals from groups purposively sampled to maximize diversity of types of carer characteristics from: (1) carers who have used the ATT for at least 6 months, (2) carers who have requested ATT withdrawal after installation and (3) carers who have refused ATT when offered. To achieve theoretical saturation (the point at which no new themes emerge), at least ten participants will be interviewed from each group, with up to a further five from each if needed [43].

### Statistics

#### Sample size calculation

On average, 50% of participants with a BADLS score of >15 will enter residential care after 24 months [44]. A reduction in the estimated 24-month transition to care home rate by 30% (i.e. 50% institutionalised at 2 years reduced to 35%) would require the involvement of 500 participants allowing for 10% attrition due to death while still living in a community setting. This equates to an average of 55 days of longer independent home life for participants receiving ATT. The trial would therefore be powered to detect a mean institutional delay of a little less than 8 weeks. Expert opinion suggests that 8 weeks is close to the minimum clinically important difference in delaying institutionalisation [45].

#### Analyses

Statistical analysis will be by intention to treat (ITT). All participants who are randomised will be included in the comparison and analysed according to their randomised allocation, including those who discontinue the ATTILA study. Wherever possible, we will continue to collect follow-up data from these participants after they leave the study, so that the data set will be as complete as possible.

The primary outcome of time to institutionalisation will be analysed using standard log rank methods and will include all events, even those occurring after 2 years. Continuous outcome measures, e.g. health-related quality of life and functional ability measures, will be analysed using repeated measures regression techniques to maximise statistical power. Exploratory subgroup analyses will be undertaken, with appropriate caution, to investigate any influence of the baseline prognostic factors (gender, age, risk profile, support structure) on outcome.

For qualitative data, thematic analysis of transcripts and field notes will be undertaken to develop themes emerging inductively from data and narrative analysis of longitudinal elements to identify and contextualise acceptable and less-acceptable practices and features of AT equipment use. A collaborative analytic strategy will involve appropriate research team members and service user/carer researchers to enhance validity and to help integrate qualitative and quantitative findings. This component will be subject to a supplementary application for ethical review.

Local, site-specific reports will be produced by each local ATTILA research site towards the end of the fieldwork period. The timing of reports and locally specific content will be locally discussed and negotiated between the local PI and designated senior managers within the local CASSR.

##### Handling of potential bias and heterogeneity across centres

Since there may be some heterogeneity in the practice of the research workers in identifying ATT needs across the different centres, between-centre heterogeneity will be explored in stratified analyses.

Because ATTILA is not a blinded study, it is conceivable that participants in the control group may be offered ATT by CASSR staff because they believe that risks to safety or independence might be better managed in this way, to delay or prevent institutionalisation. As a safeguard against such potential outcome bias, we will collect detailed data on ‘cross-overs’ in the control arm and reasons for this: in particular whether the cross-over is due to high risk incidents and the introduction of ATT is seen as an alternative to institutional care. To identify such potential outcome bias we will undertake sensitivity analyses, counting such participants as equivalent to meeting the primary outcome (i.e. equivalent to institutionalisation). However, the design of the study is pragmatic and the primary analysis will be restricted to actual cases of institutionalisations as awareness that the patient is, or is not, receiving these technologies will be just one of the complex factors that underlie institutionalisation.

##### Handling of missing data

Reasons for withdrawing from the study will be collected and, since stopping the ATTILA intervention is likely to be informative (e.g. a failure of or intolerance to the intervention), this information will be used in sensitivity analyses to investigate and reduce the impact of missing data.

##### Handling of reports of death

It is likely that a substantial number of deaths may occur amongst participants who have been previously institutionalised and therefore will have met the primary outcome. For deaths among participants resident in the community, deaths that occur as a consequence of an identified risk that the ATT might have affected will be included as a secondary outcome measure (documented as a serious adverse event) and the number of identified un-related deaths in the two study groups will be compared in the overall analysis and included in a sensitivity analysis of the primary outcomes.

##### Competing risk analysis

At baseline, we will collect data on the categories of risk (e.g. safety within the home or wandering) and the recommended ATT intervention to protect against this risk. In cases where two types of technology are recommended that might both reduce a particular risk, we will undertake competing risk analyses to try to disentangle the relative efficacy of each intervention. We will enhance statistical power for such analyses by reviewing each institutionalised patient to classify the contribution of particular risk behaviours to the participant’s transition to institutional care.

### Ethical considerations

There are important ethical issues to be considered in any research involving people who are living with a diagnosis of dementia.

#### Ethics and randomised controlled trial (RCT) design

Although there is some research evidence to suggest ATT may have benefits, most of this evidence is based on small-scale and qualitative studies. Whilst much of this evidence supports the use of assistive technologies in maintaining safety in some specific cases and situations, it is insufficient to support conclusions that effectiveness and utility of ATT have been established. For this reason, we consider that it is ethical to conduct a trial as we have proposed here, to provide more definitive evidence about the impact of ATT on the lives of people with dementia and their carers.

##### Seeking consent

We will assume that consent is not an ‘event’ but a process. We will therefore actively seek reaffirmed consent on all the occasions that we contact participants and seek their data.

##### Release of personal data by the CASSR

We will, depending on the wish of the CASSR, either agree to a data exchange protocol to allow for the exchange of personal data or introduce a simple protocol to the FACS assessment procedures in the CASSR over the recruitment period. Social work practitioners will ask newly referred, and eligible, people for their permission for their contact details to be released to us so that we can contact them.

##### Informed consent

It will be made clear to potential participants and their carers what taking part in the study will entail, that they are not obliged to take part in the trial and that the care that they will continue to receive from the NHS and local CASSR teams will not be adversely affected if they choose not to participate or wish to withdraw from the trial at any point.

##### Mental capacity

A diagnosis of dementia does not necessarily mean that the potential participant cannot consent to take part in the study and careful, but tactful checks will be made to establish their capacity to consent. When the person does not have capacity to consent, we will seek to identify someone known to the participant who would be prepared to act as the participant’s consultee, within the meaning of England’s Mental Capacity Act (2005).

##### Case accountability

Legal responsibility for the provision of services will rest at all times with the host CASSR in each study site. We will record the type and amount of care services, care support and ATT provided, but we will not intervene in the way in which these services are deployed (unless our advice is sought by the host CASSR). We recognise the concerns of some participants, their carers and staff about the ethics of withholding ATT, particularly as in some sites, ATT is being actively promoted as beneficial. CASSR staff will therefore, in practice, retain the right to suggest, for example, that participants in either the intervention or control group be admitted into nursing or residential care, or, in the case of control participants, recommend ATT if they feel that without it, the person’s ability to remain living safely in the community would be jeopardised. The research team will monitor for adverse incidents and will act to withdraw any participant if they assess that it is not in the best interests of the participant to continue in the study. The Independent Data Monitoring Committee (IDMC) will meet to review all adverse events’ data together with effectiveness and efficacy data at least every 6 months and will recommend discontinuing the trial prematurely if it becomes apparent from primary outcome data that efficacy has been established or that the frequency of adverse events indicates that effectiveness of the intervention is overwhelmingly likely.

##### Participant benefit

In practice, participants in studies of this kind benefit from taking part as involvement can produce positive effects. Our objective, however, is to produce evidence that will provide a robust basis for service development and will therefore benefit people living with dementia and their carers. The study team has the appropriate skills and experience in conducting clinical trials with people with dementia and includes those with specific expertise in the use and evaluation of ATT in dementia, statistics, health economics evaluation and social research methodologies. The team is distributed over a number of large population centres, which will enable the recruitment of the relatively high numbers of participants that the trial design requires. Large-scale clinical trials are expensive to conduct but are needed to provide the definitive answers that important clinical questions required so as to develop and implement clinically and cost-effective interventions within the NHS and CASSRs in England.

## Discussion

ATT is promoted by the Department of Health in England as a means of supporting older people at home. ATT is increasingly being offered as a mainstream service, and investment in this technology by CASSRs is increasing rapidly in the belief that it will save money. At present there is limited evidence to support this view. The only existing RCT evidence (from the WSD) did not specifically include people with dementia. The needs of this group mean that ATT will need to be deployed differently, and the impact of ATT may also be different compared to people without dementia. At a time of economic austerity, the provision of ATT may not, in fact, be cost-effective. If, however, ATT is shown to help people with dementia remain independent and improve their health-related quality of life, as well as being cost-effective, then investment in ATT will be fully justified.

## Trial status

In progress.

## Abbreviations

ATT: Assistive technology and telecare; CASSR: Councils with Adult Social Services Responsibilities; NHS: National Health Service; FACS: Fair Access to Care Services; RCT: Randomised controlled trial; WSD: Whole system demonstrator.

## Competing interests

A list of investigators with competing interests and the details that they declared is as follows:

Dr Iracema Leroi received research funding, speaker and consultancy fees from companies associated with dementia care, including Novartis, Pfizer, Eisai, Shire, Boehringer-Ingleheim and Lundbeck.

Dr John Woolham received travelling and accommodation expenses from Tunstall PLC to speak at a conference in Madrid in 2006.

Professor John O’Brien received research funding, speaker or consultancy fees from companies associated with dementia care, including Novartis, Pfizer, Eisai, Shire, Lundbeck, Lilly, GE Healthcare, Bayer Healthcare, TauRx, Nutricia and Cytox.

Dr Chris Fox received research funding, speaker and consultancy fees from companies associated with dementia care, including Naturica, Novartis, Pfizer, Eisai, Shire, Boehringer-Ingleheim and Lundbeck.

Professor Martin Knapp received research funding or consultancy fees from companies associated with dementia care, including Janssen and Lundbeck.

The following authors declare that they have no competing interests: Ms Rebecca Gathercole, Professor Robert Howard, Dr Peter Bentham, Dr Craig Ritchie, Professor Alistair Burns, Dr Rupert McShane, Professor Richard Gray, Ms Barbara Dunk, Professor Kirsty Forsyth, Professor Stanton Newman, Dr Anna Davies and Dr Fiona Poland.

## Authors’ contributions

IL and JW were part of the protocol development team and drafted the manuscript. RG revised the paper. RH, BD, CF, JO, MK and FP assisted in the preparation of the manuscript. PB, CR, AB, RM, RG, MK, KF, SN and AD have contributed to the design of the trial and other written materials that this manuscript has drawn on. All authors read the manuscript and commented on and approved the final manuscript.
